# COVID-19 Vaccination Intention in Patients with Autoimmune Diseases in Indonesia: An Application of the Integrated Behavioural Model

**DOI:** 10.3390/tropicalmed8020109

**Published:** 2023-02-09

**Authors:** Alvina Widhani, Dicky C. Pelupessy, Tommy Hariman Siddiq, Sukamto Koesnoe, Suzy Maria, Evy Yunihastuti, Ghina Shabrina Awanis, Teguh Harjono Karjadi, Anshari Saifuddin Hasibuan, Nanang Sukmana, Mulki Hakam, Kartika Qonita Putri, Insy Nafisah Taufik, Delina Widiyanti, Iris Rengganis, Samsuridjal Djauzi

**Affiliations:** 1 Allergy and Clinical Immunology Division, Department of Internal Medicine, Faculty of Medicine, Universitas Indonesia/Cipto Mangunkusumo Hospital, Jakarta 10430, Indonesia; 2 Faculty of Psychology, Universitas Indonesia, Depok 16424, Indonesia; 3 Faculty of Psychology and Education, Universitas Al-Azhar Indonesia, Jakarta 12110, Indonesia; 4 Antam Medika Hospital, Jakarta 13210, Indonesia

**Keywords:** patients with autoimmune diseases, COVID-19 vaccine, integrated behavioral model

## Abstract

Vaccine hesitancy can be a challenge for those with autoimmune diseases. This study investigated the acceptance of COVID-19 vaccination by patients with autoimmune diseases in Indonesia using the integrated behavioral model (IBM). This cross-sectional study was conducted from December 2021 to February 2022. A total of 404 patients with autoimmune diseases completed the survey. The majority of respondents (57.9%) said they intended to get vaccinated against COVID-19. The IBM model with added demographic variables explained 54.1% of the variance of vaccination intention (*R*^2^ = 0.541). Self-efficacy, perceived norms, experiential attitude, and instrumental attitude are significantly correlated with vaccination intention in components of health behavior theories. Self-efficacy is the most critical factor influencing vaccination intention in patients with autoimmune diseases (F(2, 401) = 96.9, *p* < 0.001, *R*^2^ = 0.326). In the multivariate analysis, vaccine intention was found to be positively associated with patients’ occupation as health-care workers (β = 0.105). Meanwhile, having a personal history of contracting COVID-19 and having co-morbidities other than autoimmune diseases were negatively correlated to the willingness to be vaccinated against COVID-19. This study confirms the viability of the IBM model for predicting the COVID-19 vaccination intention of patients with autoimmune diseases. It is essential to provide patients with autoimmune diseases with information that is clear and supported by evidence-based medicine.

## 1. Introduction

More than two years have passed since the outbreak of the coronavirus disease-2019 (COVID-19). The COVID-19 pandemic is still causing an unprecedented global crisis. Vaccines appear to be the most promising option for combating the virus and protecting people who may be more vulnerable to severe COVID-19, such as those with autoimmune diseases [1]. Vaccination of a large proportion of the population is necessary for establishing herd immunity. However, vaccine hesitancy, or the unwillingness or refusal to be vaccinated, has emerged as a serious global public health concern, particularly because it may impede the attainment of herd immunity status. In the population of patients with autoimmune diseases, vaccine hesitancy would make them prone to severe COVID-19 infection.

Patients suffering from autoimmune diseases are particularly vulnerable to COVID-19 infection. The heterogeneity of autoimmune diseases and treatment with diverse immunomodulators might result in immunocompromised patients. As a result, the vulnerability to virus infection increases and severe disease can result. Compared to the general population, the attitudes of patients with autoimmune diseases regarding vaccination and immunological reaction may differ [2,3].

Only a few studies were conducted on vaccine willingness in autoimmune patients. One study showed that the willingness to take the COVID-19 vaccine was worse in patients with autoimmune diseases than in the control group from the general population [2]. Behavioral health models have not been used in most studies examining particular concerns and factors associated with COVID-19 vaccine intention in patients with autoimmune diseases. Vaccine hesitancy can be an obstacle for people with autoimmune diseases [2,3]. Therefore, understanding the underlying drivers of vaccine hesitancy in the COVID-19 pandemic is essential from a public-health standpoint.

Not many studies have been conducted on COVID-19 vaccination intention in Indonesia, especially in distinct vulnerable populations. Analyzing the behavior of the population under study should help determine which components are most likely to influence COVID-19 vaccine intention. The integrated behavioral Model (IBM), also known as the integrative model, is a framework for recognizing difficulties and developing message and behavior-change tactics [4,5,6].

The integrative model combines many prominent theories of health behavior—such as the theory of reasoned action (TRA)/theory of planned behavior (TPB), and the health belief model (HBM) [6]. Individuals can develop vaccination behavior if they have a strong desire to become vaccinated, are not restricted in their ability to do so by their environment, understand the necessity of vaccination behavior, and have a history of doing so [6]. This study is aimed to validate the IBM model as a means for predicting COVID-19 vaccination intention in patients with autoimmune disorders in Indonesia.

In order to increase public trust and understanding about vaccination programs, especially in specific groups such as patients with autoimmune diseases, sound and transparent communication based on scientific evidence is needed. Considering the pivotal role of the vaccine in this pandemic, experts and stakeholders must understand the use of the behavior framework on approved COVID-19 vaccines. Hence, this research is also aimed at investigating the acceptance of COVID-19 vaccination in patients with autoimmune diseases in Indonesia.

## 2. Materials and Methods

### 2.1. Theoretical Framework

The integrated behavioral model (IBM), also called the integrative model (IM), is the product of expanding the theory of reasoned action (TRA); theory of planned behavior (TPB); and other prominent behavioral theories by Montaño, Fishbein, and colleagues. TRA, TPB, the health belief model (HBM), and the social cognitive theory [4] are all included in the integrated behavioral model (IBM). The first construct in the model is behavior. Behaviour is defined as a single observable action performed by an individual. As in the TRA/TPB, the most important determinant of behavior is the intention to perform the behavior, and there should be a high degree of correspondence between intention and behavior. According to the model, behavioral intention is mainly determined by the three theoretical constructs (attitude, perceived norm, and personal agency) [4,6,7].

Attitude is composed of affective and cognitive dimensions. Affect, or ‘experiential attitude’, is the individual’s emotional response (feelings) to the idea of performing a recommended behavior. The cognitive dimension is determined by ‘instrumental attitude’, which is ruled by beliefs about the outcomes of behavioral performance. Together, experiential attitude and instrumental attitude determine the attitude displayed toward the behavior. In this study, both experiential attitude and instrumental attitude form the patients’ attitude toward having to vaccinate against COVID-19 [4,6].

Perceived norm is divided into ‘injunctive norm’ and ‘descriptive norm’. Injunctive norm is defined as normative beliefs about what others think one should do. Descriptive norm is the perceptions about what others in the individual’s social or personal networks are doing. This construct captures the social identity in certain cultures and the distinct characteristics of a particular population [6].

In IBM, personal agency is represented by two constructs, namely ‘perceived control’ and ‘self-efficacy’. Perceived control is heavily determined by one’s perception of how various factors make it easy or difficult to carry out the behavior. On the other hand, self-efficacy is someone’s degree of confidence in performing the behavior in the face of challenges or barriers [6,7].

Finally, other demographic, personality, and individual difference characteristics may be related to behaviors; however, their influence is thought to be indirect and to work through the theoretical constructs. These are referred to as distal variables. As a result of demographic disparities in the proximal variables, certain demographic groups may be more likely than others to engage in a certain behavior [6].

To design effective treatments to alter behavioral intentions, it is first necessary to assess the extent to which attitude (experiential and instrumental), perceived norm (injunctive and descriptive), and personal agency (self-efficacy and perceived control) influence the intention. Once this is established for a particular behavior and population, knowing the determinants (specific beliefs) of these constructs is critical [6,7].

### 2.2. Study Design

This cross-sectional study was conducted from December 2021 to February 2022. The subjects of this study were patients with autoimmune diseases in Indonesia. The exclusion criteria were patients being unwilling to follow the study or not completely filling out the online survey.

The study was divided into two phases: (1) a qualitative research-elicitation phase to identify concerns related to COVID-19 vaccination among a representative sample of patients with autoimmune diseases, and (2) a cross-sectional quantitative survey of patients with autoimmune diseases.

Elicitation interviews were conducted as part of formative research to prepare the survey. Individual qualitative interviews, structured by IBM components, were conducted in local language with ten patients. Participants were asked to think about getting a COVID-19 vaccine; and then, describe feelings and beliefs about outcomes, sources of normative influence, and barriers and facilitators for getting the vaccine. Content analysis of the transcribed interviews yielded lists of feelings, behavioral outcomes, sources of normative influence about getting the vaccine, and barriers and facilitators to getting the vaccine. Twenty patients were then invited to help formulate the survey instrument by picking the most significant components of norms, attitude, and beliefs. This process generated the final survey instrument.

The final survey instrument also included sections about sociodemographic characteristics. Data was collected using an online survey through Google Form in Bahasa Indonesia. Information about the study was broadcast online through autoimmune patient support groups, flyers, and social media. The online consent form included a disclaimer stating that participation was voluntary and no penalties were involved for refusing to participate. This study was conducted in accordance with the Declaration of Helsinki and was approved by the Ethics Committee of the Faculty of Medicine, Universitas Indonesia.

### 2.3. Survey Instrument

The authors developed the instrument used in this study. For all the IBM constructs, items were evaluated on a 7-point bipolar scale, from strongly disagree (1) to strongly agree (7).

Behavioral intentions were measured using three items related to patients’ intention to get the COVID-19 vaccine: “I hope to be able to vaccinate against COVID-19 when a schedule is available”, “I want to vaccinate against COVID-19 with the currently available vaccine”, and “I will continue to vaccinate against COVID-19 even though there are obstacles to doing so”. Scores were averaged to constitute a measure of intention: scores above 5 on a scale from 1 to 7 were categorized as “intends to get the vaccine”, scores between 3 and 5 were categorized as “unsure”, and scores below 3 were categorized as “does not intend to get the vaccine”.

This study used an indirect measure to calculate the constructs. An indirect measure of how a person feels about performing a behavior is calculated by multiplying the person’s belief about each outcome by the rating for that outcome and then, adding up all of the product scores for all of the behavior outcomes. For instance, a person may believe that ‘getting a vaccine’ is very unlikely to result in ‘infertility’ (belief scored as 1) and may evaluate ‘infertility’ as very bad (evaluation scored as 1), resulting in a belief x evaluation product score of 1. Beliefs and evaluations of all the salient outcomes of this behavior enter the computation of an indirect measure of the person’s attitude.

Thirteen instrumental attitude and experiential attitude (affect) questions were used to assess attitude toward the behavior. These were multiplied by a comparable item assessing the outcome evaluation. All the multiplicative scores were added together across all objects. Eight descriptive normative items were used to assess perceived norms, which were multiplied by a comparable item assessing identification with the referent. Injunctive norms were not assessed in this study because this construct was not identified during the elicitation phase. Three control belief items were used to assess self-efficacy, which were multiplied by a perceived power item. Furthermore, three control belief items were used to assess perceived control, which were multiplied by a perceived power item.

### 2.4. Data Analysis

Data were analyzed using SPSS^®^ Statistics 25 software (IBM Corp., Armonk, NY, USA). Descriptive analyses were carried out on background or sociodemographic characteristics. Mean and standard deviation are reported for continuous variables. Percentages are reported for categorical variables. Bootstrapping was performed to address non-normality within the linear model framework and achieve statistical conclusion validity. Following this, analyses were conducted to identify specific beliefs underlying the IBM constructs that best explained COVID-19 vaccination intention and might be the ideal targets for intervention messages. Hierarchical multiple regression was performed with the enter method on each of the IBM constructs and sociodemographic variables significantly associated with intention.

## 3. Results

### 3.1. Background Characteristics

**Figure 1:**
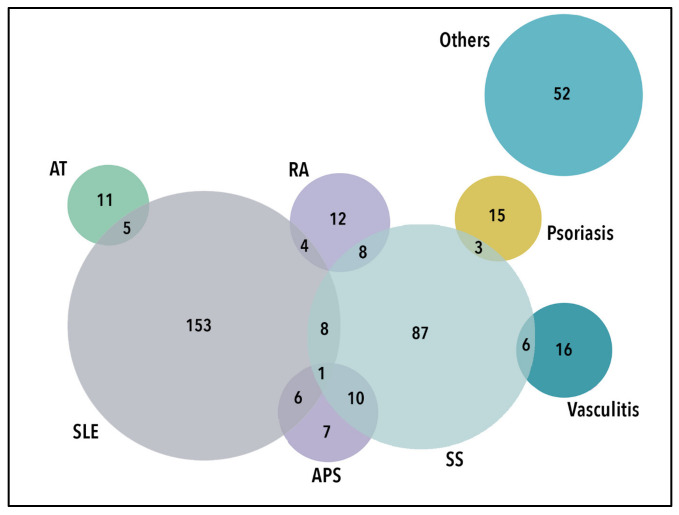
Venn diagram of participants’ autoimmune diagnosis (N = 404). AT: autoimmune thyroiditis; RA: rheumatoid arthritis; SLE: systemic lupus erythematosus; SS: Sjogren’s syndrome; APS: anti-phospolipid syndrome.

A total of 433 responses were recorded, of which 13 (3.2%) were removed due to insufficient data or data inconsistency, and 16 (3.9%) were removed due to doubled data. Thus, data from 404 patients with autoimmune diseases were analyzed. The most common diagnosis reported was systemic lupus erythematosus (SLE) (43.8%), followed by Sjogren’s syndrome (30.4%). Twenty percent of patients reported more than one autoimmune disease. The details of patient diagnoses are depicted in Figure 1.

The vast majority of respondents (57.9%) said they intended to get vaccinated against COVID-19 (scores above 5 on a scale from 1 to 7). In all, 6.7 percent of participants said they would not be vaccinated (scores below 3) and 35.4 percent said they were unsure (scores between 3 and 5).

The respondents’ average age was 38 years, with a standard deviation (SD) of 11.3. About 89.1% of the respondents were female. More than two-thirds of the respondents (73%) had a bachelor’s degree education level or higher. In addition, 27.2 percent of the patients stated that they were already vaccinated against COVID-19. Additionally, 29.2% of respondents admitted that they had already contracted COVID-19 (Table 1).

### 3.2. Final Regression Model

The constructs and the associated items used in this study were assessed for their reliability. All the constructs yielded from the elicitation process were found to have a high Cronbach’s alpha value (Table 2).

The model consisted of the constructs of the integrative model (Table 3, Model 1) and showed 51.4% variance in vaccine intention in the autoimmune patient population (*R*^2^ = 0.514). Factors significantly correlated with vaccination intention among the constructs of health behavior theories are self-efficacy, perceived norms, experiential attitude, and instrumental attitude. The most significant construct influencing vaccine intention in this study is self-efficacy. Further analysis using multiple linear regression demonstrated that the items of self-efficacy had a substantial collective influence (F(2, 401) = 96.9, *p* < 0.001, *R*^2^ = 0.326). The items ‘I have confidence that I can be vaccinated against COVID-19′ (t = 12.673, *p* <0.001) and ‘Unclear flow and procedure to access health facilities affected me getting vaccinated against COVID-19′ (t = 2.418, *p* = 0.016) were the most significant predictors in the model when individual predictors were further investigated.

Items of the perceived norm and influences of other people collectively influenced vaccine intention (F(4, 399) = 94.535, *p* < 0.001, *R*^2^ = 0.487). The people who were highly influential on patients in this study were family (t = 6.382, *p* <0.001), government programs (t = 5.172, *p* <0.001), supervising doctors (t = 3.978, *p* <0.001), and close friends (t = 2.624, *p* = 0.009).

Additionally, the combined items of experiential attitude also had a significant effect (F(3, 400) = 19.3, *p* < 0.001, *R*^2^ = 0.126). When the individual items were investigated further, the most significant predictor in the model was ‘I’m worried the COVID-19 vaccine will affect the medicines I’m taking’ (t = −4.771, *p* <0.001). Other items that had a significant effect on intention were ‘I’m worried about the long-term side effects of vaccination’ (t = 2.912, *p* = 0.004) and ‘I’m worried about getting vaccinated against COVID-19 because it might not result in good immunity because I have an autoimmune condition’ (t = −2.667, *p* = 0.008).

The results of the multiple linear regression also revealed that the constructs of instrumental attitude had a combined significant effect (F(3, 400) = 79.188, *p* < 0.001, *R*^2^ = 0.373). The significant predictors in the model were ‘Getting vaccinated against COVID-19 is one way to end the pandemic’ (t = 3.56, *p* < 0.001), ‘Getting vaccinated against COVID-19 will reduce the severity of SARS-CoV-2 infection’ (t = 2.777, *p* = 0.006), and ‘Getting vaccinated against COVID-19 means I’m contributing to herd immunity’ (t = 2.672, *p* = 0.008). The results from the stepwise regression of each construct are available in Table S1 (Supplementary File).

When the demographic characteristics are added into the model, the constructs and the demographic characteristics showed 54.1% variance in vaccination intention (*R*^2^ = 0.541). In the multivariate analysis, vaccine intention is positively associated with the occupation of health-care worker. Meanwhile, having a personal history of contracting COVID-19 and having co-morbidities other than autoimmune diseases are negatively correlated to the willingness to be vaccinated against COVID-19.

## 4. Discussion

The intention to get vaccinated against COVID-19 among the population of patients with autoimmune diseases was examined in this study. To the authors’ knowledge, this is the first study that describes patients with autoimmune diseases’ intention to be vaccinated against COVID-19 in Indonesia. According to our findings, more than half of respondents (57.9%) intended to be vaccinated against COVID-19. However, 6.7 percent of individuals indicated they would not be vaccinated, while 35.4 percent were doubtful. This finding is similar to previous studies [2,8,9], and does not differ much from findings among the general population [2,9]. However, it is much lower than a study by Correa-Rodriguez et al., which found that 96.7 percent of respondents planned to be COVID-19 vaccinated [10].

Several different COVID-19 vaccines are available in Indonesia, including AstraZeneca/Oxford (recombinant ChAdOx1 adenoviral vector encoding the spike protein antigen of the SARS-CoV-2), Moderna (mRNA-based vaccine encapsulated in a lipid nanoparticle (LNP)), Pfizer/BioNTech (modified nucleoside mRNA), Sinopharm (inactivated), Sinovac (inactivated), and Covovax (recombinant nanoparticle prefusion spike protein formulated with Matrix-M™ adjuvant) [11]. In Indonesia, the COVID-19 vaccine is given based on availability. Moreover, patients with specific comorbidities, including patients with autoimmune diseases, need to show a letter of recommendation from their physician. These conditions could influence the patients’ perceived control and other behavioral constructs.

This study indicated that incorporating health behavior constructs as an IBM model can explain Indonesian patients with autoimmune diseases’ intention to be vaccinated against COVID-19. The IBM model used in this study indicated that 51.4% of the patients intended to get the COVID-19 vaccine (*R*^2^ = 0.514). Moreover, the model with added demographic variables showed a 54.1% variance in vaccination intention (*R*^2^ = 0.541). This number is higher than our previous study on Indonesian health workers, which used the same method (*R*^2^ = 0.42) [12]. This finding is also comparable to previous studies on the general population, which integrate health behavior theories to predict COVID-19 vaccine acceptance [13].

The findings of this study suggest that the factors significantly correlated with vaccination intention among the constructs of health behavior theories are self-efficacy, perceived norms, experiential attitude, and instrumental attitude. In particular, self-efficacy was found to have the most significant influence on patients’ intention.

Self-efficacy, one of the key constructs in many health behavior theories, is defined as the belief or confidence that one can perform a recommended behavior [6]. The most significant item in this construct is ‘I have confidence that I can be vaccinated against COVID-19′. It is speculated that patients with autoimmune diseases rely more on their own beliefs regarding COVID-19 vaccination rather than physician recommendations or external factors. In line with this finding, perceived control, which relies heavily on external factors, is not significantly associated with COVID-19 vaccination intention. This finding differs from our earlier study on health-care workers in Indonesia, where perceived control was considered a significant driver of COVID-19 vaccine acceptance. Clear and transparent information based on evidence needs to be delivered to overcome misinformation that may affect patients’ beliefs [14].

This finding could also be correlated to the fact that patients with autoimmune diseases who are willing to be vaccinated can access COVID-19 vaccinations easily in the hospitals where they usually receive care. Patients feel a higher sense of self-ability to gain COVID-19 vaccination. Intervention methods should cater to this unique characteristic in patients with autoimmune diseases in order to solidify the belief of being able to be vaccinated. Previous studies have reported that higher self-efficacy in chronic patients can be attained through effective patient–physician interaction [15]. Reducing anxiety in unsure patients and verbal reinforcement from social circles can be utilized to influence self-efficacy in the population of patients with autoimmune diseases [6]. In addition to the patient confidence, this study indicates that unclear flow and procedure to access health facilities affect the intention of COVID-19 vaccination. These need to be addressed by health-care authorities, and the information about the procedure must be clearly disseminated.

Interestingly, respondents in this study still rely on the opinions of their closest ones when it comes to getting a COVID-19 vaccine. This is shown by the most significant item in the perceived norm being ‘My family expects me to get vaccinated against COVID-19′. The next most significant items were government programs and recommendations of the supervising doctor. From this result, it is clear that educating the patients and their families is vital. Furthermore, this finding re-emphasizes the need for clear and transparent information, especially from the government or health care authorities.

Among this study’s patients with autoimmune diseases, individual variables positively associated with COVID-19 vaccination intention included being employed as a health-care worker (HCW). Having a double burden of a higher risk of COVID-19 infection, as a HCW and a patient, must have increased the intention to be vaccinated. These patients also have a better risk perception of COVID-19 infection. However, the prevalent fear of side effects in this particular population should also be addressed. A study in the HCW population in Italy showed that vaccine hesitation in the HCW population was substantially related to the fear of vaccination side effects and inversely related to confidence in the vaccine’s safety and efficacy [16].

Meanwhile, it was found that having comorbidities and a personal history of COVID-19 infection are negatively correlated with the intention to vaccinate against COVID-19. The latter finding has also been reported in a previous study that included 651 patients with autoimmune diseases [8]. This might be due to the common belief that being infected with COVID-19 provides enough immunity against COVID-19 reinfection. These issues need to be addressed in the education given to the patients.

In line with previous findings, experiential attitude items, such as the fear of long-term effects of vaccination and the worry that COVID-19 vaccines may affect their medical therapy, were found to be significant predictors of vaccine intention. This finding was also reported in a study involving 3104 autoimmune inflammatory rheumatic diseases (AIIRD) patients in China [17] and a global study in 29 countries [18]. A global survey from 102 countries also highlighted patients’ concerns about the safety and efficacy of the COVID-19 vaccine, and the risk of flares [19]. An observational study evaluating the safety of the two-dose regimen of BNT162b2 mRNA among AIIRD patients showed no evidence of significant disease flares six weeks after the second vaccine dose. Most adverse events were transient and mild; however, long-term follow-up data are still needed [20]. Another survey of AIIRD patients receiving inactivated COVID-19 vaccines revealed that 10.5% of respondents reported flares, and 3.5% of patients required treatment escalation [21]. In this study, patients with autoimmune diseases were also concerned that they would not develop good immunity from the COVID-19 vaccine because of their condition. A study by Ferri et al. observed lower neutralizing antibody levels in patients with systemic autoimmune diseases compared to a control group. A higher percentage of non-responders was observed in patients treated with glucocorticoid, mycophenolate mofetil, or rituximab [22]. In most patients, the benefits of the COVID-19 vaccine outweigh its harm. Unfortunately, how to best maximize vaccine-related benefits is still unknown because of varying underlying health conditions and treatments. It is recommended that patients and healthcare providers have a shared decision-making process on COVID-19 vaccination [23]. Transparent and evidence-based information is essential.

This study has limitations. As this study used an online form to collect data, patients who did not have access to the internet, elderly patients, and those who struggled with technology could not participate. As a result, this study population has a high education level and might not represent the general population of patients with autoimmune diseases in Indonesia. The survey was also sent to support groups for specific autoimmune diseases. Hence, the proportion of diseases found in the patients who participated in this study might not reflect the actual population. Moreover, vaccination intention does not always reflect actual vaccination behavior. Those who intended to be vaccinated might have encountered barriers to getting the COVID-19 vaccine, while those who were unsure might have eventually been persuaded and vaccinated.

## 5. Conclusions

This study demonstrates the feasibility of the IBM model in prediction of the intention of patients with autoimmune diseases to get the COVID-19 vaccination. Self-efficacy, perceived norms, experiential attitude, and instrumental attitude are significantly correlated with vaccination intention among the components of health behavior theories. Self-efficacy is the most critical factor influencing vaccination intention. The majority of patients with autoimmune diseases expressed concern about the long-term impact of vaccination and COVID-19 vaccinations on their medical treatment. Providing patients with autoimmune diseases with information that is transparent and supported by evidence-based medicine is vital.

## Figures and Tables

**Table 1:** Respondent characteristics (N = 404).

Variables	Estimates, n (%)
Sex	
Female	360 (89.1)
Male	44 (10.9)
Age [mean, (SD)]	38 (11.3)
Marital status	
Married	267 (66.1)
Other (single, widowed)	137 (33.9)
Education	
High school/less than high school	109 (27)
Bachelor’s degree/higher	295 (73)
Occupation	
Healthcare worker	43 (10.6)
Employee, non-HCW	139 (34.4)
Entrepreneur	62 (15.3)
Unemployed	160 (39.6)
Healthcare facility used	
Government-run (*Puskesmas*, state hospitals)	198 (49)
Others	206 (51)
Number of autoimmune diagnoses	
One	320 (79.2)
More than one	84 (20.8)
Autoimmune diagnosis	
SLE	177 (43.8)
Non-SLE	227 (56.2)
Has comorbidities	
Yes	213 (52.7)
No	191 (47.3)
Already vaccinated against COVID-19	
Yes	110 (27.2)
No	294 (72.8)
Personal history of COVID-19 infection	
Yes	118 (29.2)
No	286 (70.8)

**Table 2:** IBM theoretical constructs associated with COVID-19 vaccination intention.

Constructs and Associated Items	Corrected Item—Total Correlation	Cronbach’s Alpha (α)
*Instrumental attitude*		0.906
Getting vaccinated against COVID-19 means I provide protection for my family	0.644	
So that the pandemic ends, I will continue to follow the health protocols after/if I have been vaccinated against COVID-19	0.507	
Getting vaccinated against COVID-19 means I’m protecting myself	0.748	
Getting vaccinated against COVID-19 is one way to end the pandemic	0.85	
Getting vaccinated against COVID-19 means I’m contributing to herd immunity	0.834	
If I have COVID-19, my symptoms will be more severe than those of people without an autoimmune disease	0.669	
By getting the COVID-19 vaccine, I will have immunity against COVID-19 infection	0.810	
Getting vaccinated against COVID-19 will reduce the severity of SARS-CoV-2 infection	0.644	
*Experiential attitude*		0.605
I’m worried about getting vaccinated against COVID-19 because it might not result in good immunity because I have an autoimmune condition	0.388	
I’m worried about the long-term side effects of vaccination	0.335	
I’m worried about side effects after the COVID-19 vaccination	0.396	
I’m worried the COVID-19 vaccine will affect the medicines I’m taking	0.397	
I’m worried that my autoimmune disease will become worse after the COVID-19 vaccination	0.355	
*Perceived norms*		0.907
I am not willing to be vaccinated against COVID-19 because other patients with autoimmune diseases in the same community are not vaccinated against COVID-19	0.635	
Having friends who advised me to get vaccinated made me want to get vaccinated against COVID-19	0.743	
I got vaccinated against COVID-19 because I followed a government program	0.725	
Colleagues expect me to get vaccinated against COVID-19	0.794	
I am willing to be vaccinated against COVID-19 if the doctor who treats my condition suggests it	0.511	
My immediate supervisor expects me to get vaccinated against COVID-19	0.733	
Religious leaders I respect expect me to get vaccinated against COVID-19	0.739	
My family expects me to get vaccinated against COVID-19	0.727	
*Perceived control*		0.783
I am willing to be vaccinated if I get a COVID-19 vaccine that has been scientifically proven to have a good success rate	0.674	
News of vaccine success rates in other countries influenced my decision to be vaccinated against COVID-19	0.552	
I will vaccinate against COVID-19 if I obtain clear information about the side effects of my autoimmune disease	0.662	
*Self-efficacy*		0.616
Unclear flow and procedure to access health facilities affected me getting vaccinated against COVID-19	0.323	
If I want to, it is easy for me to get vaccinated against COVID-19	0.503	
I have confidence that I can be vaccinated against COVID-19	0.458	

**Table 3:** Final regression model.

Variables	*B*	*SE B*	β	*R*	*R* ^2^
Model 1				0.717	0.514
Self-efficacy	0.008 *	0.002	0.155		
Perceived norms	0.007 *	0.001	0.406		
Experiential attitude	−0.006 *	0.002	−0.137		
Instrumental attitude	0.005 *	0.001	0.217		
Perceived behavior control	0.001	0.002	0.015		
Model 2				0.735	0.541
Self-efficacy	0.008 *	0.002	0.154		
Perceived norms	0.006 *	0.001	0.373		
Instrumental attitude	0.005 *	0.001	0.205		
Experiential attitude	−0.005 *	0.002	−0.117		
Perceived behavior control	0.001	0.002	0.032		
Job (health-care worker)	0.474 *	0.179	0.105		
Personal history of COVID-19 infection (yes)	−0.219 *	0.11	−0.072		
Having co-morbidities (yes)	−0.21 *	0.097	−0.075		
Job (employee)	0.98	0.123	0.033		
Job (entrepreneur)	0.211	0.151	0.055		
Sex (female)	−0.202	0.163	−0.045		
Education (high)	0.95	0.126	0.03		
Marital status (married)	−0.036	0.111	−0.012		
Age	0.002	0.005	0.018		
Payment method (national health insurance [BPJS] only)	0.149	0.129	0.053		
Health facility (government-run only)	−0.202	0.127	−0.073		
Autoimmune diagnosis (more than one)	−0.127	0.122	−0.037		

* *p* < 0.05.

## Data Availability

The data used are available from the corresponding author upon request.

## Supplementary Materials

The following supporting information can be downloaded at: https://www.mdpi.com/article/10.3390/tropicalmed8020109/s1, Table S1: Further analysis on items from each IBM constructs.
