# Supplementary material for: COVID-19 Vaccination Intention in Patients with Autoimmune Diseases in Indonesia: An Application of the Integrated Behavioural Model
Source: Trop Med Infect Dis. 2023 Feb 9;8(2):109. doi: 10.3390/tropicalmed8020109 (PMC9967126; doi:10.3390/tropicalmed8020109)
Supplement: Supplementary file 1 [file tropicalmed-08-00109-s001.zip › tropicalmed-2128868-supplementary.pdf]

Table S1. Further analysis on items from each IBM constructs.

Items	r*	β (p value)	R	R^2
Instrumental attitude			0.61	0.373
Getting vaccinated against COVID-19 is one way to end the pandemic	0.581	0.276 (<0.001)		
Getting vaccinated against COVID-19 will reduce the severity of being infected with the SARS-CoV-2 virus	0.409	0.178 (0.006)		
Getting vaccinated against COVID-19 means I'm contributing to herd immunity	0.568	0.204 (0.008)		
Item(s) that did not enter stepwise regression				
Getting vaccinated against COVID-19 means I provide protection for my family	0.409			
So that the pandemic ends, I will continue to follow the health protocol after/if I have been vaccinated against COVID-19	0.276			
Getting vaccinated against COVID-19 means I'm protecting myself	0.469			
If I have COVID-19, the symptoms will be more severe than for people without an autoimmune disease	0.49			
By taking the COVID-19 vaccine, I will have immunity against COVID-19 infection	0.54			
Experiential attitude			0.356	0.126
I'm worried the COVID-19 vaccine will affect the medicines I'm taking	-0.31	-0.254 (<0.001)		
I'm worried about the long-term side effects of vaccination	0.091	0.138 (0.004)		
I'm worried about getting vaccinated against COVID-19 because it doesn't mean that good immunity is formed due to an autoimmune condition	-0.244	-0.143 (0.008)		
Item(s) that did not enter stepwise regression				

I feel worried about the side effects after the COVID-19 vaccination	N.S.			
I'm worried that my autoimmune disease will get worse after the COVID-19 vaccination	N.S.			
Perceived norms			0.698	0.487
My family expects me to get vaccinated against COVID-19	0.609	0.308 (<0.001)		
I vaccinated against COVID-19 because I followed a government program	0.595	0.259 (<0.001)		
I am willing to be vaccinated against COVID-19, if the doctor who treats my condition suggests it	0.483	0.168 (<0.001)		
Having friends who advised me to get vaccinated made me intend to get vaccinated against COVID-19	0.517	0.124 (0.009)		
Item(s) that did not enter stepwise regression				
I am not willing to be vaccinated against COVID-19 because other autoimmune patients in the same community are not vaccinated against COVID-19	0.434			
Colleagues expect me to get vaccinated against COVID-19	0.502			
My immediate supervisor expects me to get vaccinated against COVID-19	0.509			
Religious leaders I respect expect me to get vaccinated against COVID-19	0.479			
Perceived control			0.495	0.245
I will vaccinate against COVID-19 if I get clear information about the side effects of my autoimmune disease	0.324	0.187 (<0.001)		
News of vaccine success rates in other countries influenced my decision to be vaccinated against COVID-19	0.469	0.373 (<0.001)		
Item(s) that did not enter stepwise regression				

I am willing to be vaccinated if I use a COVID-19 vaccine that has been scientifically proven to show a good success rate	0.378			
Self-efficacy			0.571	0.326
I have confidence that I can vaccinate against COVID-19	0.562	0.537 (<0.001)		
Unclear flow and procedure to gain access to health facilities affected me in getting vaccinated against COVID-19	0.237	0.102 (0.016)		
Item(s) that did not enter stepwise regression				
If I want to, then it's easy for me to get vaccinated against COVID-19	0.327			

* $p < 0.05$ for all correlations, except N.S. (not significant).
